# Cutaneous Metastases Without a Known Primary: A Clinical Conundrum

**DOI:** 10.7759/cureus.83591

**Published:** 2025-05-06

**Authors:** Dhairya Gor, Bipin Ghimire, Omar Abbas, Maria Diab

**Affiliations:** 1 Hematology and Oncology, Henry Ford Health System, Detroit, USA; 2 Pathology and Laboratory Medicine, Henry Ford Health System, Detroit, USA; 3 Internal Medicine, Michigan State University, Lansing, USA

**Keywords:** adenocarcinoma, carcinoma of unknown primary, cutaneous metastases of unknown primary, diagnostic and therapeutic challenge, solitary skin metastases

## Abstract

Cutaneous metastases of unknown primary are a relatively rare clinical entity, presenting unique diagnostic and management challenges despite advancements in diagnostic techniques. Due to their rarity, management often requires an individualized, multidisciplinary approach. These metastases are typically associated with a poor prognosis. Clinicians should consider this diagnosis when evaluating skin lesions, as early recognition is crucial for optimal outcomes. Here, we present the case of a 56-year-old male with a skin lesion on his left leg, highlighting the diagnostic complexities and management strategies associated with cutaneous metastases of unknown primary.

## Introduction

Cutaneous metastases are a rare occurrence in oncology, reported to have an incidence of less than 1% in various studies [1]. Melanoma is the most frequent source of these metastases, followed by breast and lung cancers [2]. Skin metastases can be diagnosed synchronously (at the same time as the primary cancer), metachronously (after the initial diagnosis), or, very rarely, as the first indication of malignancy [2-3]. Carcinoma of unknown primary (CUP) is defined as a carcinoma or undifferentiated neoplasm where a standardized diagnostic workup fails to identify the primary tumor [4]. Typical presentation sites for CUP include the liver, respiratory system, lymph nodes, abdominal cavity, bones, and brain [5]. When cutaneous metastases occur as the sole manifestation of malignancy without an identified primary site, it becomes even rarer within this already uncommon entity [5]. We present a case of cutaneous metastasis of unknown primary, with a potential lower gastrointestinal (GI) origin, in which no primary lesion was identified through imaging or endoscopic evaluation.

## Case presentation

**Figure 1:**
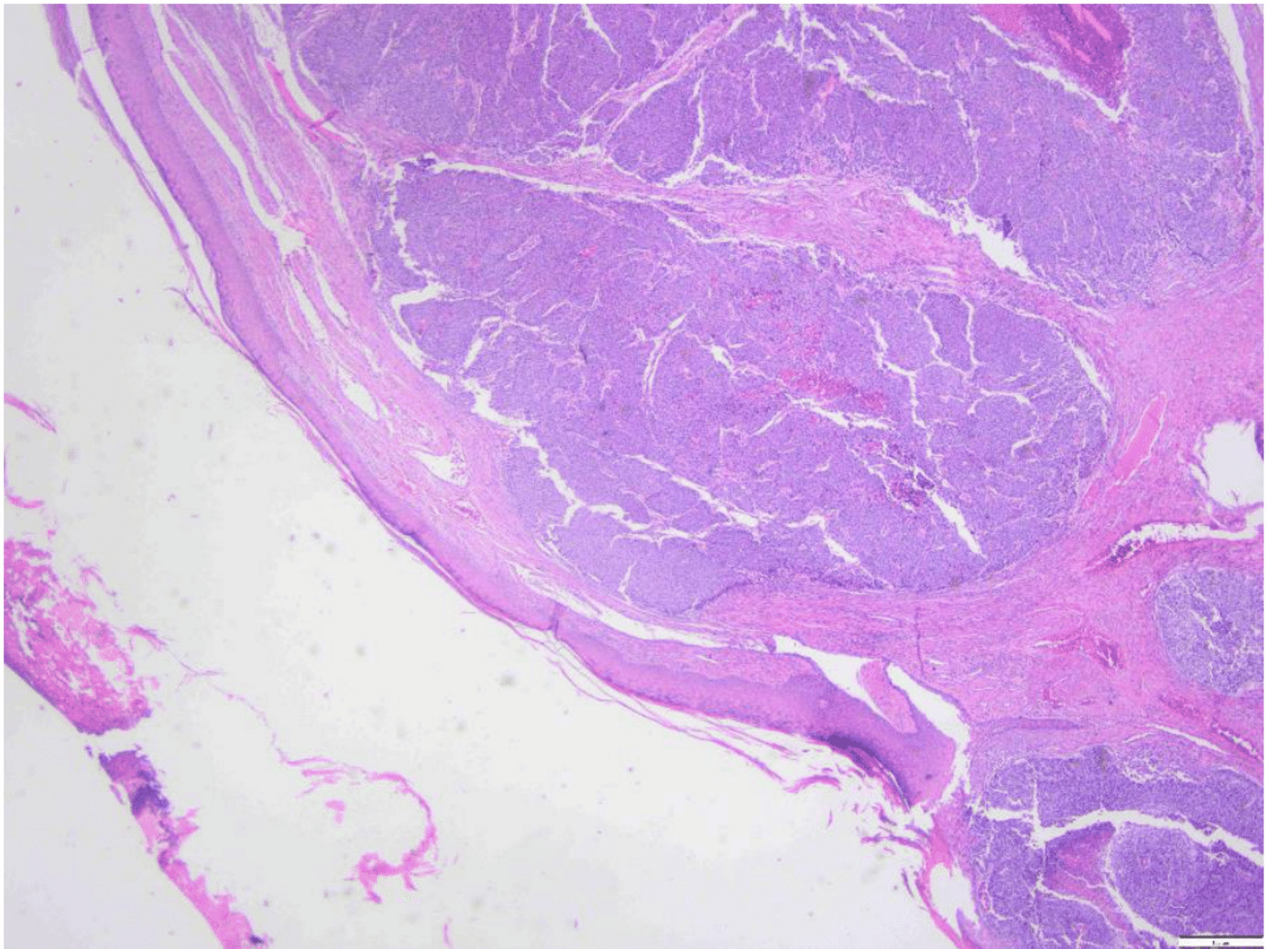
Low-power magnification (40x) H&E section of the lesion shows lobules of neoplastic glandular cells located within the dermis. The overlying epithelium is ulcerated but does not exhibit any in situ lesions. This favors a metastatic lesion.

**Figure 2:**
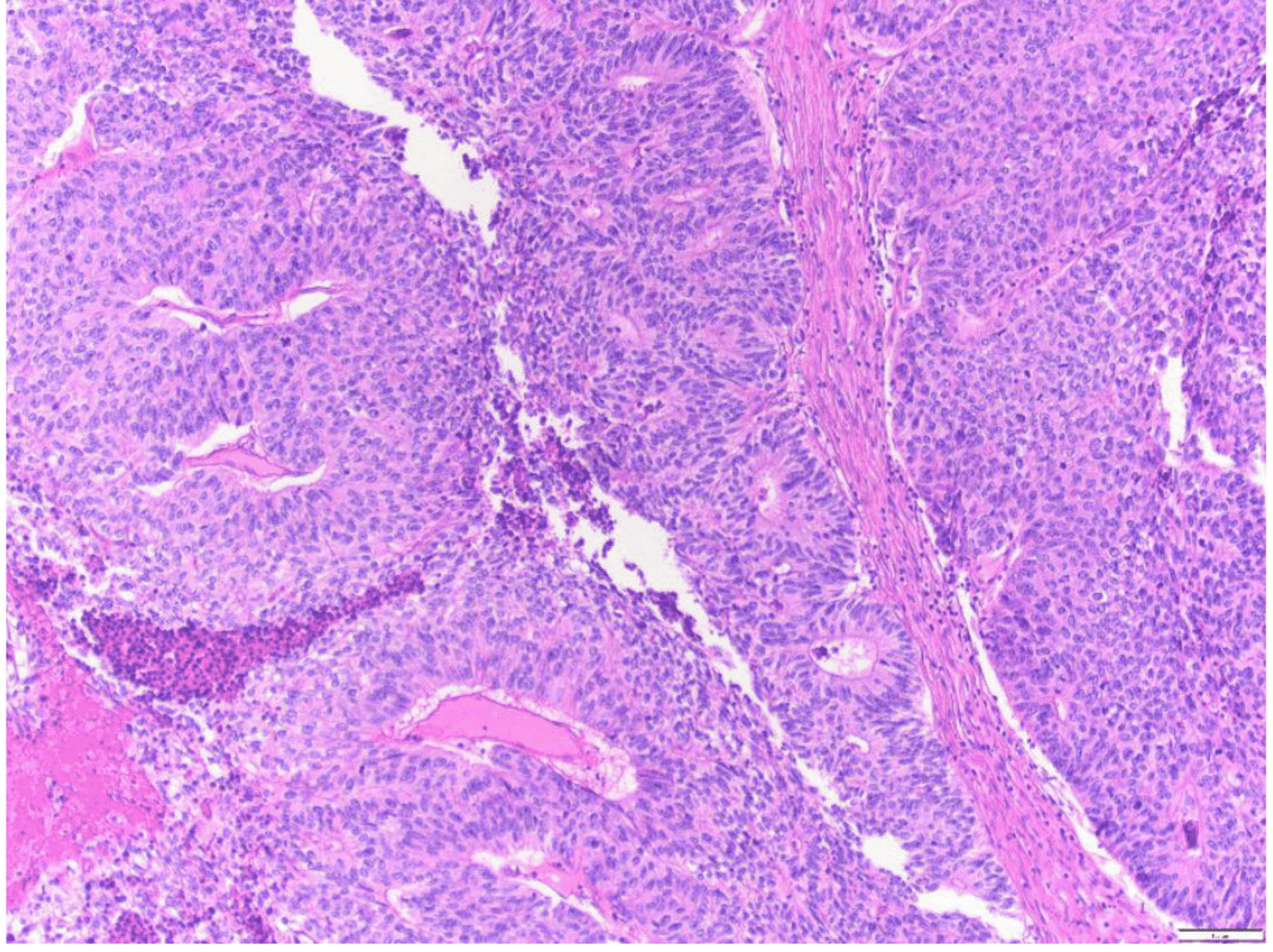
Higher magnification (100x) H&E demonstrating neoplastic glandular cells in the dermis with solid and cribriform type architecture.

A 56-year-old male with a history of familial adenomatous polyposis (FAP) presented with a painful skin lesion on his left knee and upper leg, which had developed spontaneously over 4 to 5 weeks. The lesion progressively enlarged and bled upon touch, prompting medical evaluation. He was afebrile, the remainder of the physical examination was unremarkable, and routine laboratory tests, including a complete blood count, coagulation parameters, renal function tests, and liver function tests, were within normal limits. Initially, the lesion was suspected to be a pyogenic granuloma. Surgical excision of the lesion revealed pathology consistent with adenocarcinoma of unknown primary. Histological examination showed lobules of glandular neoplastic cells in the dermis, characterized by solid and cribriform architectures (Figure 1, Figure 2).

**Figure 3:**
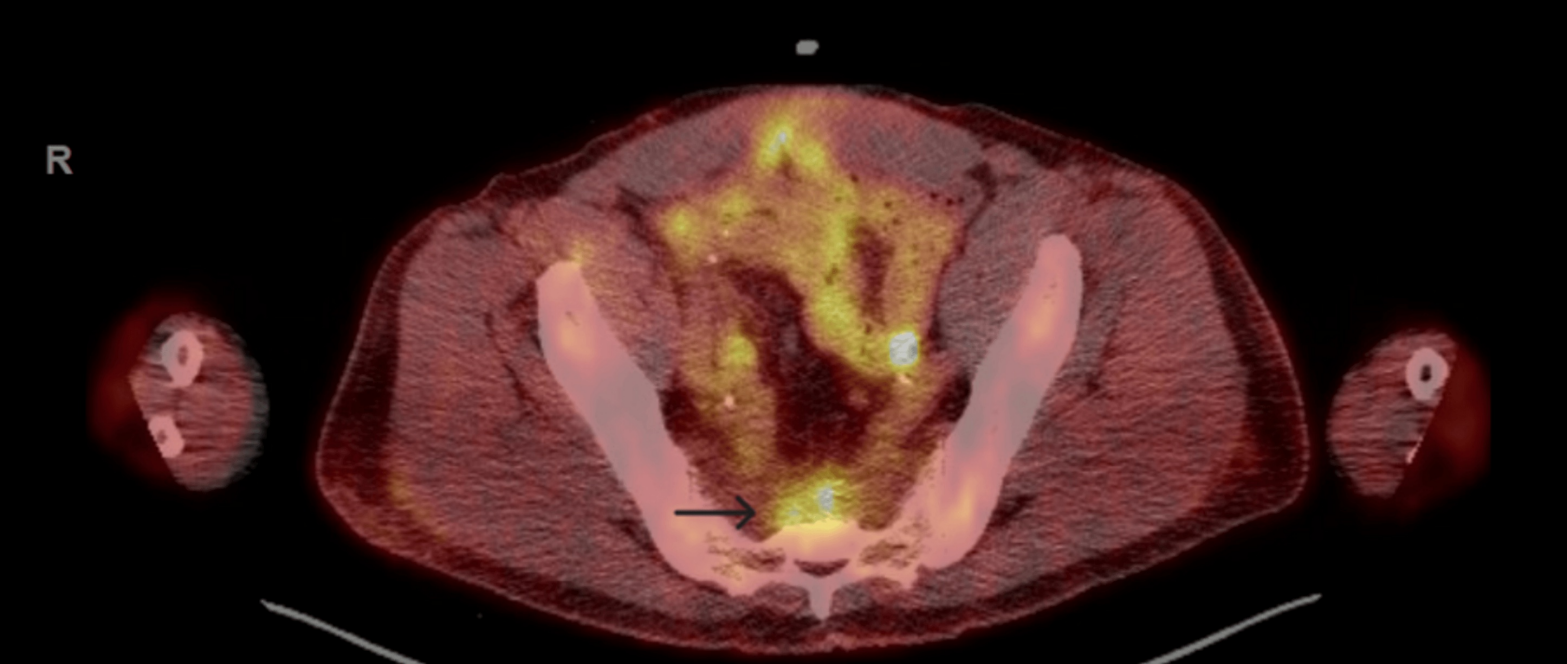
PET scan showing presacral mass-like opacity with intense increased radiotracer uptake, SUV max 7 SUV max: maximum standardized uptake value

The overlying epithelium was ulcerated but did not show in situ lesions. Immunohistochemistry (IHC) demonstrated positivity for AE1/AE3, CK7, CK20 (patchy positivity), CDX2, CK5/6 (with foci of negativity), CD56 (focal), p40 (focally positive), TTF-1 (patchy positivity), synaptophysin (focally positive) and chromogranin (positive in rare cells), INSM-1 (positive in rare cells), beta-catenin, and PAX8 (focally positive), but was negative for GATA3, Napsin A, NKX3.1, and SOX10. The peripheral and deep margins were positive for residual tumor. While a lower gastrointestinal origin was suspected based on the morphology and IHC, a specific diagnosis could not be established. The patient had been diagnosed with FAP nine years earlier, at age 47, and had undergone total colectomy, with surgical pathology negative for malignancy. Given his FAP history and the adenocarcinoma's likely intestinal origin, endoscopic evaluation of the remnant ileal mucosa was performed but revealed no masses or lesions. Carcinoembryonic antigen (CEA) levels were within normal limits. Further evaluation through CT imaging of the chest, abdomen, and pelvis revealed a 6.5 x 5.7 x 5.0 cm presacral mass with indistinct borders, along with adjacent lymphadenopathy suspicious for malignancy. Subsequent PET scan showed presacral mass-like opacity with intense increased radiotracer uptake, SUV max (maximum standardized uptake value) 7 (Figure 3).

**Figure 4:**
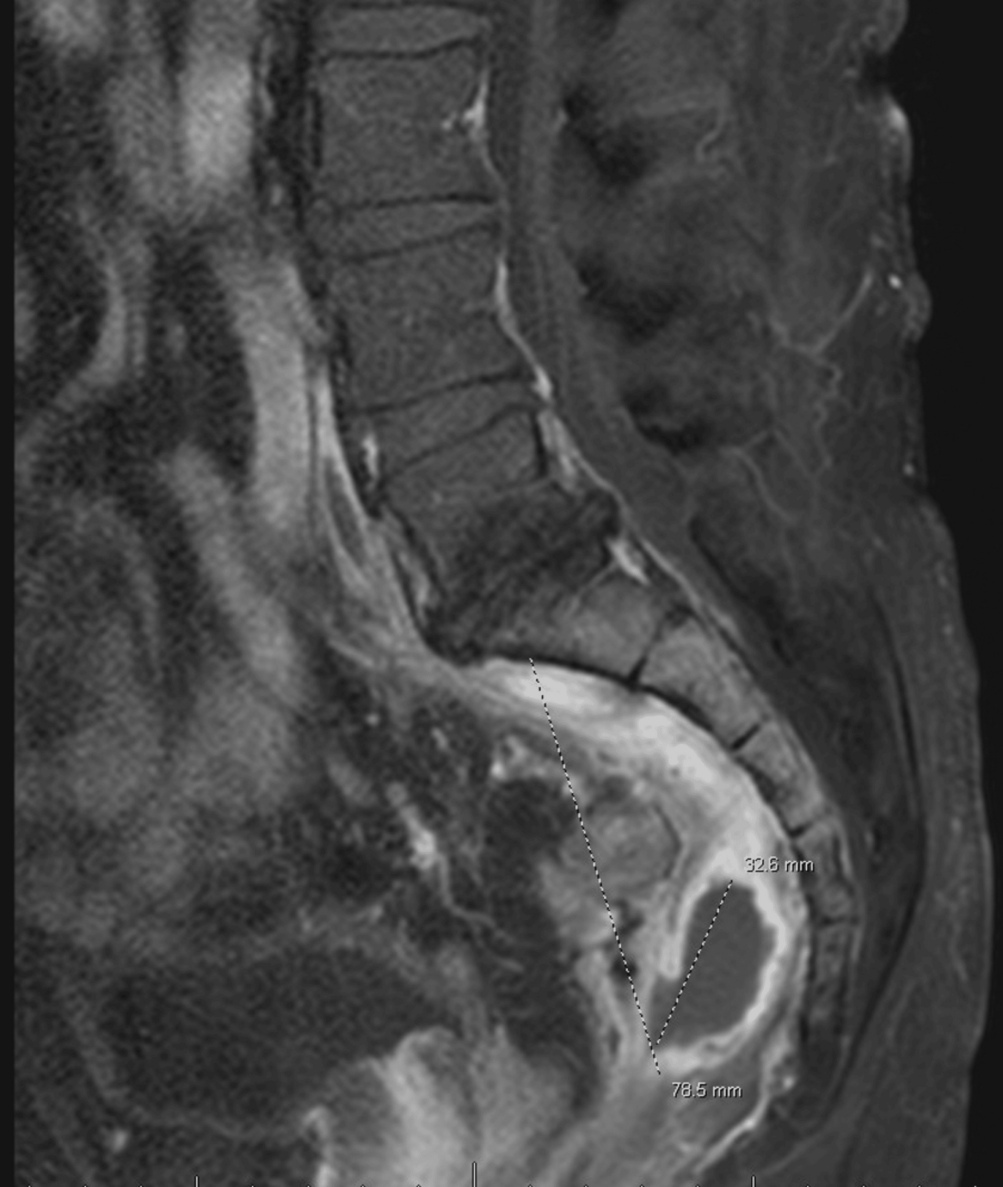
MRI sacrum showing edema and enhancement throughout the presacral region, compatible with a presacral abscess

MRI showed edema and enhancement throughout the presacral region, compatible with a presacral abscess (Figure 4).

**Figure 5:**
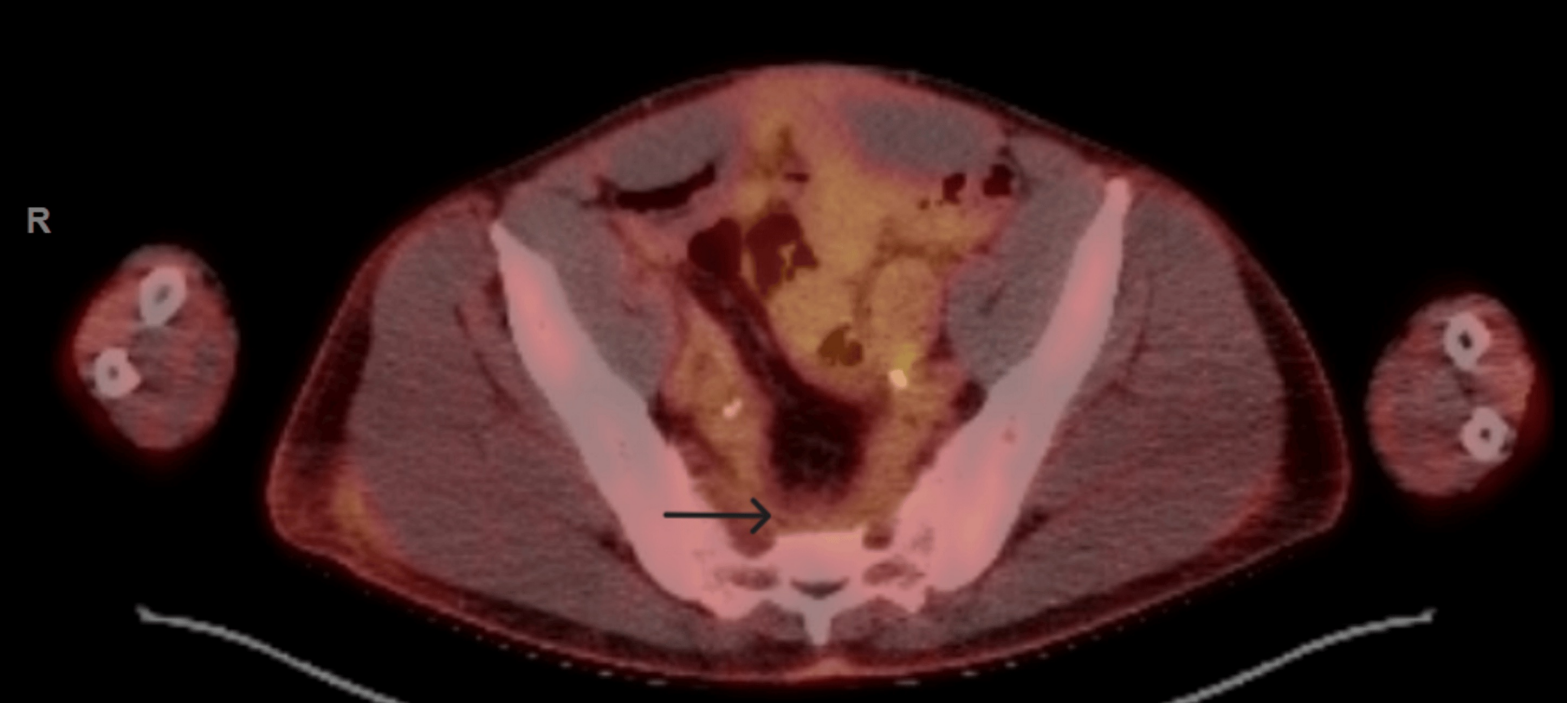
PET scan showing markedly reduced metabolic activity in the presacral mass

A CT-guided biopsy of the mass returned negative for malignant cells, but cultures were positive for *Streptococcus anginosus, *and the source of infection was thought to be a skin lesion. The patient was treated with six weeks of antibiotics (intravenous ceftriaxone and oral metronidazole). Repeat blood cultures after treatment with antibiotics were negative. Due to the absence of an identified primary site and positive surgical margins, the patient underwent radiation to the skin lesion, receiving 51 Gy in 17 fractions. Follow-up imaging with PET-CT six weeks post-radiation showed markedly reduced metabolic activity in the presacral mass, with no other avid lesions (Figure 5).

**Figure 6:**
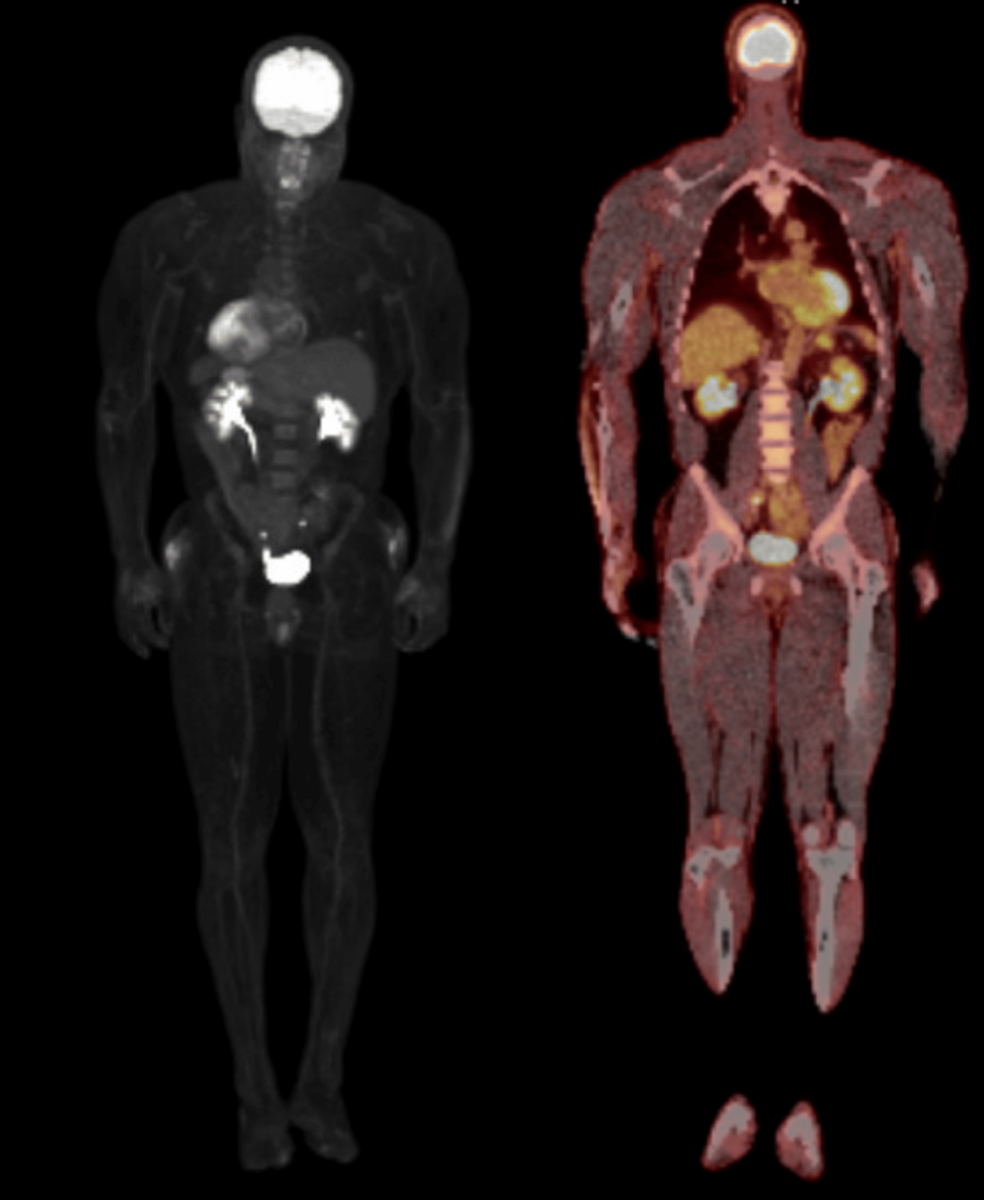
PET-CT showing no abnormal uptake

Subsequent surveillance CT scans indicated continued improvement, and a repeat PET-CT six months later showed complete resolution of radiotracer activity in the presacral region and no evidence of uptake anywhere else (Figure 6).

The most recent scans continue to show no evidence of disease activity. He has now been disease-free for 11 months since his radiation therapy and 15 months since the excision of the primary skin lesion.

## Discussion

This case highlights a unique occurrence in oncology. CUP accounts for less than 5% of cancers, and its incidence is decreasing with advancements in diagnostic techniques [5]. The specific incidence of cutaneous metastases as the only disease site in CUP is not well-documented, with literature primarily consisting of case reports and small studies. Although a primary lesion was not identified, a lower GI origin was suspected in our patient. Typically, colorectal adenocarcinoma metastasizes to the liver and lungs, with cutaneous metastases being a rare occurrence, reported in approximately 3% [6]. Our case also illustrates the diagnostic complexity in determining the origin of cancer in cutaneous metastases.

As noted by Habermehl and Ko, the diagnostic approach relies heavily on morphology and IHC [2]. The glandular morphology and positive markers like CK7, CK20, and CDX2 suggested a potential lower intestinal origin. However, the presence of other lineage markers and patchy positivity complicated the diagnosis, necessitating detailed discussion among oncologists and pathologists. Differentiating cutaneous metastases from primary skin adnexal malignancies (e.g., cutaneous apocrine, eccrine, or sebaceous carcinomas) is also crucial [5]. Certain pathological features can aid this distinction, such as tumor cell presence in lymphatic and blood vessels, substantial tumor mass in the deep dermis and subcutaneous fat, and dispersed neoplastic cells among collagen bundles [7].

Without a known primary site, management of CUP can be challenging [5,8]. Treatment options may include local interventions (radiation, surgery) or empiric systemic therapy [8]. In cases of solitary metastatic lesions, such as in our patient, local treatment may be sufficient for disease control, followed by careful monitoring [5]. The decision for localized treatment was based on the absence of additional metastatic disease and the patient’s overall good functional status. Notably, systemic chemotherapy, often a mainstay in CUP management, was deferred given the isolated nature of the metastasis and the patient’s favorable response to local therapies. 

Beyond the rarity and diagnostic complexities, this case underscores the importance of a multidisciplinary approach to management and the need for awareness among physicians of solitary skin metastasis as a potential indicator of underlying malignancy. Initially, our patient was misdiagnosed with a pyogenic granuloma based on the clinical presentation. The prognosis for CUP patients is generally poor, with median survival ranging from six to nine months, although this varies based on disease burden [7]. Despite the generally poor prognosis associated with CUP, particularly in cases with distant metastases, our patient has remained disease-free for approximately 15 months following the surgical excision of the skin lesions and 11 months post-radiation therapy. Early recognition of this entity can lead to timely diagnosis and treatment, potentially improving survival outcomes [9].

## Conclusions

This case highlights a rare presentation of CUP with solitary cutaneous metastasis. Diagnosis requires thorough evaluation, and overall management is challenging. The patient underwent surgical excision, followed by radiation therapy, with systemic treatment deferred due to the localized nature of the disease and the patient’s good functional status. The patient continues to show no evidence of disease, highlighting the potential for effective long-term control in select cases of CUP managed with aggressive local therapy. Continued surveillance is essential, as cutaneous metastases may precede the detection of an underlying malignancy by several months to years.
